# Draft Genome Sequence of Bacillus paralicheniformis Strain GSFE7, a Halotolerant Plant Growth-Promoting Bacterial Endophyte Isolated from Cultivated Saline Areas of the Dead Sea Region

**DOI:** 10.1128/mra.00425-22

**Published:** 2022-08-11

**Authors:** Randa Albdaiwi, Tareq Alhindi, Shireen Hasan

**Affiliations:** a Department of Land, Water, and Environment, School of Agriculture, The University of Jordan, Amman, Jordan; b Department of Biological Sciences, School of Science, The University of Jordan, Amman, Jordan; c Hamdi Mango Center for Scientific Research, The University of Jordan, Amman, Jordan; SIPBS, University of Strathclyde

## Abstract

Here, we report the draft genome sequence of Bacillus paralicheniformis strain GSFE7, which was isolated from saline fields near the Dead Sea region. The genome was 4,452,800 bp in size and contained 4,382 coding sequences. Several genes were predicted to be involved in auxin production, nitrogen fixation, phosphate mobilization, and putative production of siderophores and antibiotics such as bacitracin, butirosin, and fengycin.

## ANNOUNCEMENT

Bacillus paralicheniformis was first identified in 2015 (1); since then, several strains have been reported to have plant growth-promoting properties (PGPPs) (2–4). Bacillus paralicheniformis strain GSFE7 was isolated from the roots of durum wheat cultivated in saline fields near the Dead Sea in Jordan (altitude, −249 m; latitude, 31°48′52″N; longitude, 35°37′42″E) (5). The region has an arid and warm climate, with annual precipitation of <100 mm and maximum temperatures of >40°C during the summer.

Root samples were collected in sterile plastic bags and were placed on ice packs and transferred to the laboratory within 1 h. The samples were washed under running tap water for 10 min to remove adhering soil particles. The roots were disinfected for 1 min with 70% ethanol, rinsed three times with sterile distilled water, and then surface sterilized for 10 min with 3% sodium hypochlorite solution with a few drops of Tween 20, followed by six rinses with sterile distilled water. Sterilized roots (1 g) were macerated in 10 mL of 1% NaCl solution, and serial dilutions were prepared, spread on nutrient agar (NA) medium supplemented with 10% NaCl, and incubated at 28°C for 1 week for bacterial colony formation. Several isolates were selected, and single colonies were purified by subculturing three times on NA medium supplemented with 10% NaCl. Strain GSFE7 was found to possess antifungal activities against Fusarium culmorum (5).

Total DNA was isolated from a single colony using the Wizard genomic DNA purification kit (Promega), and the concentration and quality of the DNA were determined using a spectrophotometer. The DNA was sent to Macrogen Inc. (Seoul, South Korea) for sequencing using the Illumina NovaSeq 6000 platform (library type, TruSeq Nano DNA [350-bp insert]; type of reads, paired-end reads; read length, 151 bp). The quality of the raw reads (total of 23,432,396 reads) was assessed using FastQC v0.11.9 (6). Fastp v0.23.1 (7) was used to trim adaptor sequences and to remove bad reads. The sequence reads were *de novo* assembled into 24 contigs (*N*_50_, 756,180 bp; GC content, 45.55%) using SKESA v2.4.0 (8). The draft genome (total size, 4,452,800 bp, consisting of 24 contigs) was annotated using the NCBI Prokaryotic Genome Annotation Pipeline (PGAP) v6.1. A total of 4,382 candidate protein-coding genes and a total of 77 tRNA genes and 12 rRNA genes were identified. A digital DNA-DNA hybridization was performed via the Genome-to Genome Distance Calculator v3.0 implemented in the TYGS server (9), and the results showed that strain GSFE7 has 95% sequence identity to B. paralicheniformis strain Bac84 (NCBI reference sequence accession number NZ_CP023665.1), with a 0.29% difference in the GC content, thus assigning it to this species epithet. All software tools were run with default parameters unless otherwise specified.

### Data availability.

The draft genome of Bacillus paralicheniformis strain GSFE7 was submitted to the National Center for Biotechnology Information (NCBI) under BioProject accession number PRJNA822385, BioSample accession number SAMN27182028, and GenBank accession number JALJEF000000000.1. Annotation data are available under GenBank assembly accession number GCA_022953115.1.
